# Supplementary material for: COVI-Prim survey: Challenges for Austrian and German general practitioners during initial phase of COVID-19
Source: PLoS One. 2021 Jun 10;16(6):e0251736. doi: 10.1371/journal.pone.0251736 (PMC8191874; doi:10.1371/journal.pone.0251736)

S1 Fig**.** Differences between GPs in single-handed and not single-handed practices in their evaluation of the pandemic


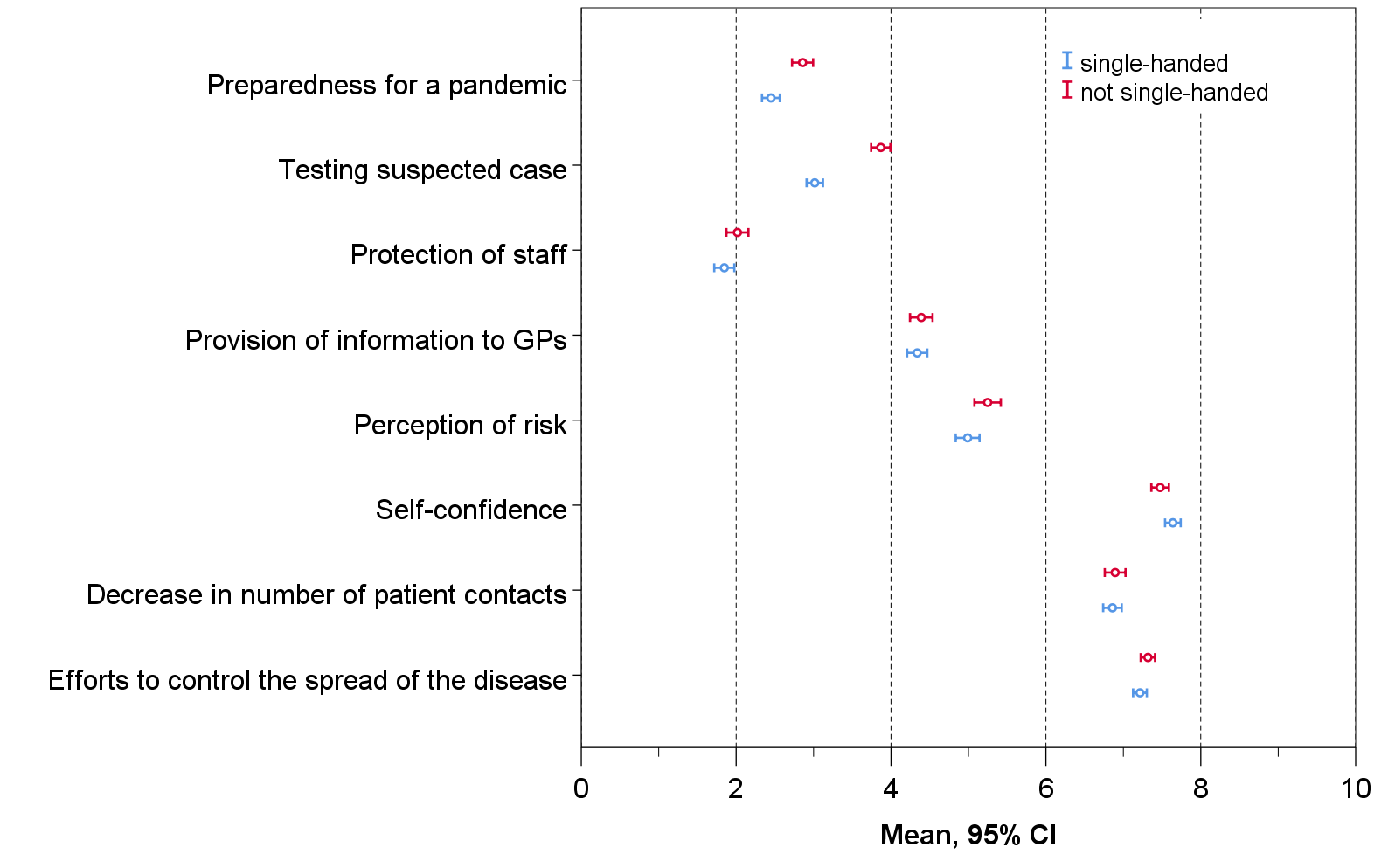

Supplement: S1 Fig — (DOCX) [file pone.0251736.s001.docx]
