# Supplementary material for: COVI-Prim survey: Challenges for Austrian and German general practitioners during initial phase of COVID-19
Source: PLoS One. 2021 Jun 10;16(6):e0251736. doi: 10.1371/journal.pone.0251736 (PMC8191874; doi:10.1371/journal.pone.0251736)
Supplement: S1 Table — (DOCX) [file pone.0251736.s002.docx]

S1 Table. Response distribution (%) for all items

|  | | no | probably  no | probably  yes | yes |  |
| --- | --- | --- | --- | --- | --- | --- |
| **Perception of risk** | | | | | | |
|  | I am worried that people I live with could catch Covid-19 from me. | 16 | 28 | 32 | 24 |  |
|  | I am afraid that I will catch Covid-19 from a patient. | 28 | 39 | 20 | 14 |  |
|  | It causes me concern that I want to care for my patients but at the same time do not want to endanger my family. | 21 | 28 | 26 | 26 |  |
|  | I am worried that I may unknowingly infect my patients. | 14 | 31 | 31 | 24 |  |
|  | My employees are worried about catching Covid-19 from patients. | 11 | 39 | 31 | 19. |  |
| **Provision of information to GPs** | | | | | | |
|  | I received guidelines on how to deal with suspected cases of Covid-19 in good time. | 13 | 27 | 41 | 19 |  |
|  | The guidelines on how to deal with suspected cases of Covid-19 were sufficiently detailed. | 12 | 28 | 41 | 19 |  |
|  | At the beginning of the Covid-19 pandemic, I received sufficient information from public bodies | 36 | 36 | 20 | 8 |  |
|  | At the beginning of the Covid-19 pandemic, I had sufficient information on how to deal with suspected cases. | 27 | 28 | 31 | 134 |  |
|  | My employees and I were easily able to contact the responsible health care authorities. | 31 | 31 | 27 | 11 |  |
|  | Important information was available to patients on public media sooner than it was officially provided to family practitioners in information letters from the responsible institutions (e.g. health insurance funds). | 41 | 30 | 21 | 8 |  |
| **Preparedness for a pandemic** | | | | | | |
|  | At the beginning of the Covid-19 pandemic, I had enough protective equipment on hand. | 74 | 14 | 8 | 4 |  |
|  | My practice was well prepared for the Covid-19 pandemic. | 43 | 34 | 17 | 5 |  |
|  | At the beginning of the Covid-19 pandemic, I knew where I could get hold of protective equipment. | 55 | 23 | 13 | 8 |  |
|  | At the beginning of the Covid-19 pandemic, I had sufficient information on how much equipment I need. | 64 | 27 | 5 | 3. |  |
|  | Currently I have enough personal protective equipment. | 24 | 25 | 31 | 20 |  |
| **Self-confidence** | | | | | | |
|  | I am convinced that I know enough to provide optimal care for my patients during the pandemic. | 3 | 15 | 52 | 30 |  |
|  | I know what to do in case of a suspected case of Covid-19. | 0 | 1 | 20 | 80 |  |
|  | When looking after patients that have been infected with Covid-19, I am sometimes unsure that I am doing everything right. | 7 | 24 | 41 | 28 |  |
| **Testing suspected cases** | | | | | | |
|  | Too little testing is being done. | 47 | 25 | 19 | 9 |  |
|  | At the beginning of the Covid-19 pandemic I had adequate access to tests (either conducted them myself. or could arrange them). | 55 | 16 | 15 | 14 |  |
|  | It would be best if all suspected cases of Covid-19 went directly to hospital so that I could look after the rest of the patients. | 7 | 10 | 27 | 57 |  |
|  | Separate hotlines should be available to enable medical personnel to arrange tests for patients. | 70 | 17 | 6 | 7 |  |
|  | We family practitioners should be able to decide who gets tested and who doesn't. | 68 | 25 | 5 | 2 |  |
| **Decrease in number of patient contacts** | | | | | | |
|  | I have less to do at the moment because many patients are not currently coming to the practice. | 10 | 14 | 32 | 44 |  |
|  | I have to look after more patients because other health care services (specialists. hospitals) are less available. | 18 | 17 | 28 | 37 |  |
|  | I have less contact to patients as a result of the pandemic. | 1 | 4 | 17 | 78 |  |
|  | I am currently treating patients that I would normally refer to specialists or to hospital. | 17 | 35 | 22 | 26 |  |
| **Efforts to control the spread of the disease** | | | | | | |
|  | I do not currently treat patients with mild illnesses that are not linked to suspected cases of Covid-19 in my practice, and attend to them by phone or online. | 6 | 6 | 34 | 54 |  |
|  | If possible, I, or one of my employees, tries to gain enough information from patients by phone in order to know whether we are dealing with a suspected case of Covid-19. | 1 | 1 | 13 | 85 |  |
|  | I use various digital channels (e.g. e-mail, WhatsApp) to share information with my colleagues so that we can support each other in the current situation. | 7 | 12 | 25 | 57 |  |
|  | I have taken precautions to ensure that suspected cases do not come into contact with other patients in my practice (e.g. separate waiting rooms, appointments at different times). | 1 | 1 | 12 | 85 |  |
|  | I contact patients that are quarantined at home in order to monitor the progression of the disease. | 19 | 12 | 22 | 46 |  |
|  | I avoid touching patients when examining them. | 28 | 26 | 33 | 13 |  |
|  | Before a patient enters my practice, he or she is screened for possible symptoms (e.g. temperature measurement). | 28 | 14 | 22 | 35 |  |
| **Protection of staff** | | | | | | |
|  | I have had to send employees home because we had too little protective equipment. | 73 | 13 | 7 | 7 |  |
|  | Some employees in my practice have ceased working since the outbreak of the Covid-19 pandemic because they belong to a vulnerable group (e.g. pregnant women, older employees). | 79 | 4 | 3 | 14 |  |
|  | I found it difficult to provide adequate information to my practice team without worrying them. | 51 | 30 | 14 | 5 |  |
| **Other items** | | | | | | |
|  | I feel helpless when I think of the patients of mine that have been infected with Covid-19. | 43 | 37 | 16 | 5 |  |
|  | I am worried about how the pandemic will affect the economic outlook of my employees and myself. | 14 | 26 | 28 | 32 |  |
|  | At the beginning of the Covid-19 pandemic, I had sufficient information on the type of personal protective equipment I need. | 42 | 26 | 19 | 13 |  |
|  | I keep a close eye on my employees and myself to see whether anyone is showing initial symptoms of an infection. | 1 | 3 | 17 | 79 |  |
|  | I have to take on patients from colleagues that have closed their practices because of quarantine. | 49 | 13 | 11 | 27 |  |
|  | I have moved out from home in order to avoid endangering my family. | 98 |  |  | 2 |  |
