# Supplementary material for: COVI-Prim survey: Challenges for Austrian and German general practitioners during initial phase of COVID-19
Source: PLoS One. 2021 Jun 10;16(6):e0251736. doi: 10.1371/journal.pone.0251736 (PMC8191874; doi:10.1371/journal.pone.0251736)
Supplement: S2 Table — Percentages were calculated as %German GPs minus %Austrian GPs. Responses which were more often chosen by German GPs are marked green and responses which were more often chosen by Austrian GPs are marked red. (DOCX) [file pone.0251736.s003.docx]

S2 Table**.** Difference in the responses of Austrian and German GPs. Percentages were calculated as %German GPs minus %Austrian GPs. Responses which were more often chosen by German GPs are marked green and responses which were more often chosen by Austrian GPs are marked red.

|  |  | no | probably  no | probably  yes | yes |
| --- | --- | --- | --- | --- | --- |
| **Perception of risk** | | | | | |
|  | I am worried that people I live with could catch Covid-19 from me. | 0 | -8 | -2 | 10 |
|  | I am afraid that I will catch Covid-19 from a patient. | 0 | -6 | 1 | 5 |
|  | It causes me concern that I want to care for my patients but at the same time do not want to endanger my family. | 2 | -5 | -2 | 6 |
|  | I am worried that I may unknowingly infect my patients. | -2 | -5 | 2 | 6 |
|  | My employees are worried about catching Covid-19 from patients. | -6 | -8 | 8 | 6 |
| **Provision of information to GPs** | | | | | |
|  | I received guidelines on how to deal with suspected cases of Covid-19 in good time. | -3 | -3 | 1 | 6 |
|  | The guidelines on how to deal with suspected cases of Covid-19 were sufficiently detailed. | -4 | -2 | 3 | 4 |
|  | At the beginning of the Covid-19 pandemic I received sufficient information from public bodies | 0 | 2 | -2 | 0 |
|  | At the beginning of the Covid-19 pandemic I had sufficient information on how to deal with suspected cases. | -1 | 1 | -1 | 0 |
|  | My employees and I were easily able to contact the responsible health care authorities. | 8 | 0 | -6 | -2 |
|  | Important information was available to patients on public media sooner than it was officially provided to family practitioners in information letters from the responsible institutions (e.g. health insurance funds). | 2 | 7 | 0 | -8 |
| **Preparedness for a pandemic** | | | | | |
|  | At the beginning of the Covid-19 pandemic, I had enough protective equipment on hand. | 1 | 1 | 3 | -5 |
|  | My practice was well prepared for the Covid-19 pandemic. | -5 | 1 | 3 | 0 |
|  | At the beginning of the Covid-19 pandemic, I knew where I could get hold of protective equipment. | -8 | 4 | 3 | 1 |
|  | At the beginning of the Covid-19 pandemic, I had sufficient information on how much equipment I need. | -1 | 0 | 0 | 0 |
|  | Currently I have enough personal protective equipment. | -4 | -2 | 1 | 5 |
| **Self-confidence** | | | | | |
|  | I am convinced that I know enough to provide optimal care for my patients during the pandemic. | 1 | 5 | -1 | -5 |
|  | I know what to do in case of a suspected case of Covid-19. | 0 | -1 | 3 | -2 |
|  | When looking after patients that have been infected with Covid-19, I am sometimes unsure that I am doing everything right. | -2 | -1 | 2 | 1 |
| **Testing suspected cases** | | | | | |
|  | Too little testing is being done. | -22 | 0 | 14 | 8 |
|  | At the beginning of the Covid-19 pandemic, I had adequate access to tests (either conducted them myself, or could arrange them). | 18 | 14 | 6 | -39 |
|  | It would be best if all suspected cases of Covid-19 went directly to hospital so that I could look after the rest of the patients. | 14 | 0 | -8 | -5 |
|  | Separate hotlines should be available to enable medical personnel to arrange tests for patients. | -20 | 6 | 5 | 9 |
|  | We family practitioners should be able to decide who gets tested and who doesn't. | -13 | 8 | 3 | 2 |
| **Decrease in number of patient contacts** | | | | | |
|  | I have less to do at the moment because many patients are not currently coming to the practice. | -4 | 6 | 2 | -3 |
|  | I have to look after more patients because other health care services (specialists, hospitals) are less available. | 14 | 4 | -7 | -12 |
|  | I have less contact to patients as a result of the pandemic. | 0 | 2 | 13 | -15 |
|  | I am currently treating patients that I would normally refer to specialists or to hospital. | -9 | -6 | 9 | 5 |
| **Efforts to control the spread of the disease** | | | | | |
|  | I do not currently treat patients with mild illnesses that are not linked to suspected cases of Covid-19 in my practice, and attend to them by phone or online. | 4 | 3 | 7 | -14 |
|  | If possible, I, or one of my employees, tries to gain enough information from patients by phone in order to know whether we are dealing with a suspected case of Covid-19. | 0 | -1 | 5 | -4 |
|  | I use various digital channels (e.g. e-mail, WhatsApp) to share information with my colleagues so that we can support each other in the current situation. | 1 | 5 | 4 | -10 |
|  | I have taken precautions to ensure that suspected cases do not come into contact with other patients in my practice (e.g. separate waiting rooms, appointments at different times). | 0 | 1 | 5 | -6 |
|  | I contact patients that are quarantined at home in order to monitor the progression of the disease. | -13 | -1 | 11 | 2 |
|  | I avoid touching patients when examining them. | 5 | 3 | -3 | -4 |
|  | Before a patient enters my practice, he or she is screened for possible symptoms (e.g. temperature measurement). | 4 | 2 | 2 | -8 |
| **Protection of staff** | | | | | |
|  | I have had to send employees home because we had too little protective equipment. | 8 | 0 | -3 | -4 |
|  | Some employees in my practice have ceased working since the outbreak of the Covid-19 pandemic because they belong to a vulnerable group (e.g. pregnant women, older employees). | -2 | 3 | 1 | -2 |
|  | I found it difficult to provide adequate information to my practice team without worrying them. | -7 | 1 | 5 | 1 |
| **Other items** | | | | | |
|  | I feel helpless when I think of the patients of mine that have been infected with Covid-19. | 1 | 0 | 1 | -1 |
|  | I am worried about how the pandemic will affect the economic outlook of my employees and myself. | -5 | -5 | 4 | 6 |
|  | At the beginning of the Covid-19 pandemic, I had sufficient information on the type of personal protective equipment I need. | -6 | -1 | 4 | 2 |
|  | I keep a close eye on my employees and myself to see whether anyone is showing initial symptoms of an infection. | 1 | 4 | 6 | -10 |
|  | I have to take on patients from colleagues that have closed their practices because of quarantine. | 8 | 1 | -1 | -8 |
|  | I have moved out from home in order to avoid endangering my family. | 1 | -1 | 0 | 0 |
