# Supplementary material for: COVI-Prim survey: Challenges for Austrian and German general practitioners during initial phase of COVID-19
Source: PLoS One. 2021 Jun 10;16(6):e0251736. doi: 10.1371/journal.pone.0251736 (PMC8191874; doi:10.1371/journal.pone.0251736)
Supplement: S3 Table — (DOCX) [file pone.0251736.s004.docx]

S3 Table**.** Differences in responses to items that do not belong to a factor (multivariable ordinal or binary logistic regression results; Bonferroni correction).

| I feel helpless when I think of the patients of mine that have been infected with Covid-19. | | | | | |
| --- | --- | --- | --- | --- | --- |
| Type of practice (p = 1.000) | | no | probably  no | probably  yes | yes |
|  | single-handed | 42,5% | 36,8% | 15,0% | 5,7% |
|  | not single-handed | 42,8% | 37,2% | 16,5% | 3,6% |
| Country of survey (p = 1.000) | |  |  |  |  |
|  | Austria | 42,1% | 37,1% | 15,3% | 5,5% |
|  | Germany | 43,0% | 36,9% | 15,9% | 4,3% |
| Size of town of practice | |  |  |  |  |
|  | < 5,000 (p = 1.000) | 38,5% | 38,9% | 17,0% | 5,7% |
|  | 5,000 - <20,000 (p = 1.000) | 42,5% | 38,0% | 15,2% | 4,4% |
|  | 20,000 - <100,000 (p = .114) | 51,7% | 29,2% | 15,0% | 4,1% |
|  | ≥100,000 (Ref.) | 41,5% | 38,7% | 15,2% | 4,6% |
| I am worried about how the pandemic will affect the economic outlook of my employees  and myself. | | | | | |
| Type of practice (p = .018) | | no | probably  no | probably  yes | yes |
|  | single-handed | 14,9% | 25,4% | 26,3% | 33,4% |
|  | not single-handed | 13,3% | 26,3% | 30,2% | 30,1% |
| Country of survey (p <.001) | |  |  |  |  |
|  | Austria | 17,0% | 29,0% | 25,4% | 28,6% |
|  | Germany | 12,3% | 23,5% | 29,7% | 34,4% |
| Size of town of practice | |  |  |  |  |
|  | < 5,000 (p = .001) | 16,9% | 30,1% | 27,9% | 25,1% |
|  | 5,000 - <20,000 (p = 1.000) | 14,6% | 25,2% | 27,3% | 32,9% |
|  | 20,000 - <100,000 (p = 1.000) | 11,9% | 20,5% | 30,4% | 37,2% |
|  | ≥100,000 (Ref.) | 12,2% | 24,7% | 27,2% | 35,9% |
| At the beginning of the Covid-19 pandemic, I had sufficient information on the type of  personal protective equipment I need. | | | | | |
| Type of practice (p = .017) | | no | probably  no | probably  yes | yes |
|  | single-handed | 44,9% | 26,4% | 17,1% | 11,6% |
|  | not single-handed | 37,4% | 25,9% | 22,7% | 14,0% |
| Country of survey (p = .756) | |  |  |  |  |
|  | Austria | 45,1% | 26,7% | 17,0% | 11,3% |
|  | Germany | 39,3% | 25,8% | 21,2% | 13,6% |
| Size of town of practice | |  |  |  |  |
|  | < 5,000 (p = 1.000) | 41,3% | 27,6% | 19,5% | 11,6% |
|  | 5,000 - <20,000 (p = 1.000) | 42,6% | 27,4% | 18,5% | 11,4% |
|  | 20,000 - <100,000 (p = 1.000) | 40,3% | 21,3% | 21,6% | 16,8% |
|  | ≥100,000 (Ref.) | 42,0% | 26,2% | 19,2% | 12,6% |

| I keep a close eye on my employees and myself to see whether anyone is showing initial  symptoms of an infection. | | | | | |
| --- | --- | --- | --- | --- | --- |
| Type of practice (p <.001) | | no | probably  no | probably  yes | yes |
|  | single-handed | 0,8% | 1,9% | 13,6% | 83,7% |
|  | not single-handed | 1,2% | 4,5% | 22,3% | 72,0% |
| Country of survey (p = .002) | |  |  |  |  |
|  | Austria | 0,6% | 0,8% | 13,6% | 85,0% |
|  | Germany | 1,2% | 4,5% | 19,8% | 74,5% |
| Size of town of practice | |  |  |  |  |
|  | < 5,000 (p = 1.000) | 0,5% | 2,1% | 16,2% | 81,2% |
|  | 5,000 - <20,000 (p = 1.000) | 1,5% | 2,7% | 17,3% | 78,5% |
|  | 20,000 - <100,000 (p = .912) | 0,9% | 3,8% | 17,0% | 78,4% |
|  | ≥100,000 (Ref.) | 1,0% | 4,0% | 18,9% | 76,1% |
| I have to take on patients from colleagues that have closed their practices because of quarantine. | | | | | |
| Type of practice (p = .046) | | no | probably  no | probably  yes | yes |
|  | single-handed | 50,1% | 12,8% | 10,7% | 26,3% |
|  | not single-handed | 47,7% | 14,3% | 11,0% | 27,0% |
| Country of survey (p <.001) | |  |  |  |  |
|  | Austria | 44,6% | 13,0% | 11,2% | 31,2% |
|  | Germany | 52,1% | 13,8% | 10,6% | 23,5% |
| Size of town of practice | |  |  |  |  |
|  | < 5,000 (p = .003) | 52,4% | 12,3% | 9,6% | 25,7% |
|  | 5,000 - <20,000 (p = .060) | 50,7% | 13,8% | 9,0% | 26,5% |
|  | 20,000 - <100,000 (p = .301) | 51,3% | 13,1% | 12,2% | 23,4% |
|  | ≥100,000 (Ref.) | 41,6% | 14,7% | 13,9% | 29,9% |
| I have moved out from home in order to avoid endangering my family. | | | | | |
| Type of practice (p = 1.000) | | no | yes |  |  |
|  | single-handed | 98,1% | 1,9% |  |  |
|  | not single-handed | 99,0% | 1,0% |  |  |
| Country of survey (p = .438) | |  |  |  |  |
|  | Austria | 97,7% | 2,3% |  |  |
|  | Germany | 99,0% | 1,0% |  |  |
| Size of town of practice | |  |  |  |  |
|  | < 5,000 (p = 1.000) | 98,3% | 1,7% |  |  |
|  | 5,000 - <20,000 (p = 1.000) | 98,3% | 1,7% |  |  |
|  | 20,000 - <100,000 (p = 1.000) | 98,8% | 1,2% |  |  |
|  | ≥100,000 (Ref.) | 98,6% | 1,4% |  |  |
