# Supplementary material for: COVI-Prim survey: Challenges for Austrian and German general practitioners during initial phase of COVID-19
Source: PLoS One. 2021 Jun 10;16(6):e0251736. doi: 10.1371/journal.pone.0251736 (PMC8191874; doi:10.1371/journal.pone.0251736)
Supplement: S2 File — (DOCX) [file pone.0251736.s006.docx]

Checklist for Reporting Results of Internet E-Surveys (CHERRIES)

| 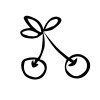 | **Checklist for Reporting Results of Internet E-Surveys (CHERRIES)** | |
| --- | --- | --- |
| ***Item Category*** | ***Checklist Item*** | ***Page*** |
| **Design** | Describe survey design | 6 |
| **IRB (Institutional Review Board) approval and informed consent process** | IRB approval | 6 |
|  | Informed consent | A-2, A-7 |
|  | Data protection | A-7 |
| **Development and pre-testing** | Development and testing | 5, A-2 |
| **Recruitment process and description of the sample having access to the questionnaire** | Open survey versus closed survey | A-5 |
|  | Contact mode | A-6 |
|  | Advertising the survey | A-6 |
| **Survey administration** | Web/E-mail | 6, A-5 |
|  | Context | A-5 |
|  | Mandatory/voluntary | A-5 |
|  | Incentives | A-5 |
|  | Time/Date | A-5 |
|  | Randomization of items or questionnaires | A-6 |
|  | Adaptive questioning | A-6 |
|  | Number of Items | 5, A-2, A-3, A-4 |
|  | Number of screens (pages) | A-3, A-4 |
|  | Completeness check | A-6 |
|  | Review step | A-6 |
| **Response rates** | Unique site visitor | NA |
|  | View rate (Ratio of unique survey visitors/unique site visitors) | NA |
|  | Participation rate (Ratio of unique visitors who agreed to participate/unique first survey page visitors) | NA |
|  | Completion rate (Ratio of users who finished the survey/users who agreed to participate) | 2,7, A-7 |
| **Preventing multiple entries from the same individual** | Cookies used | A-6 |
|  | IP check | A-6 |
|  | Log file analysis | A-6 |
|  | Registration | NA |
| **Analysis** | Handling of incomplete questionnaires | A-4 |
|  | Questionnaires submitted with an atypical timestamp | A-6 |
|  | Statistical correction | A-7 |
