# Supplementary figures and images for: COVI-Prim survey: Challenges for Austrian and German general practitioners during initial phase of COVID-19
Source: PLoS One. 2021 Jun 10;16(6):e0251736. doi: 10.1371/journal.pone.0251736 (PMC8191874; doi:10.1371/journal.pone.0251736)

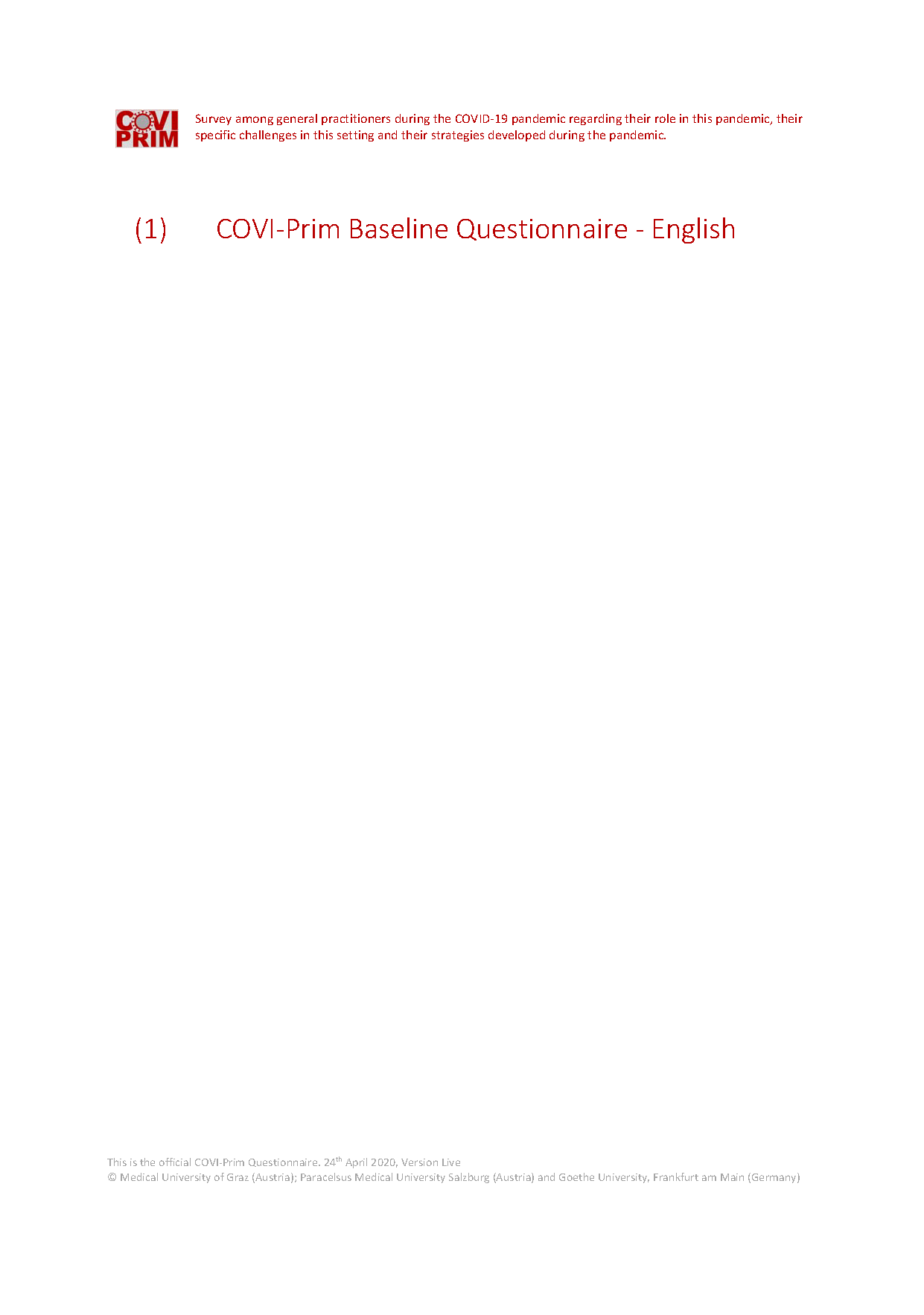

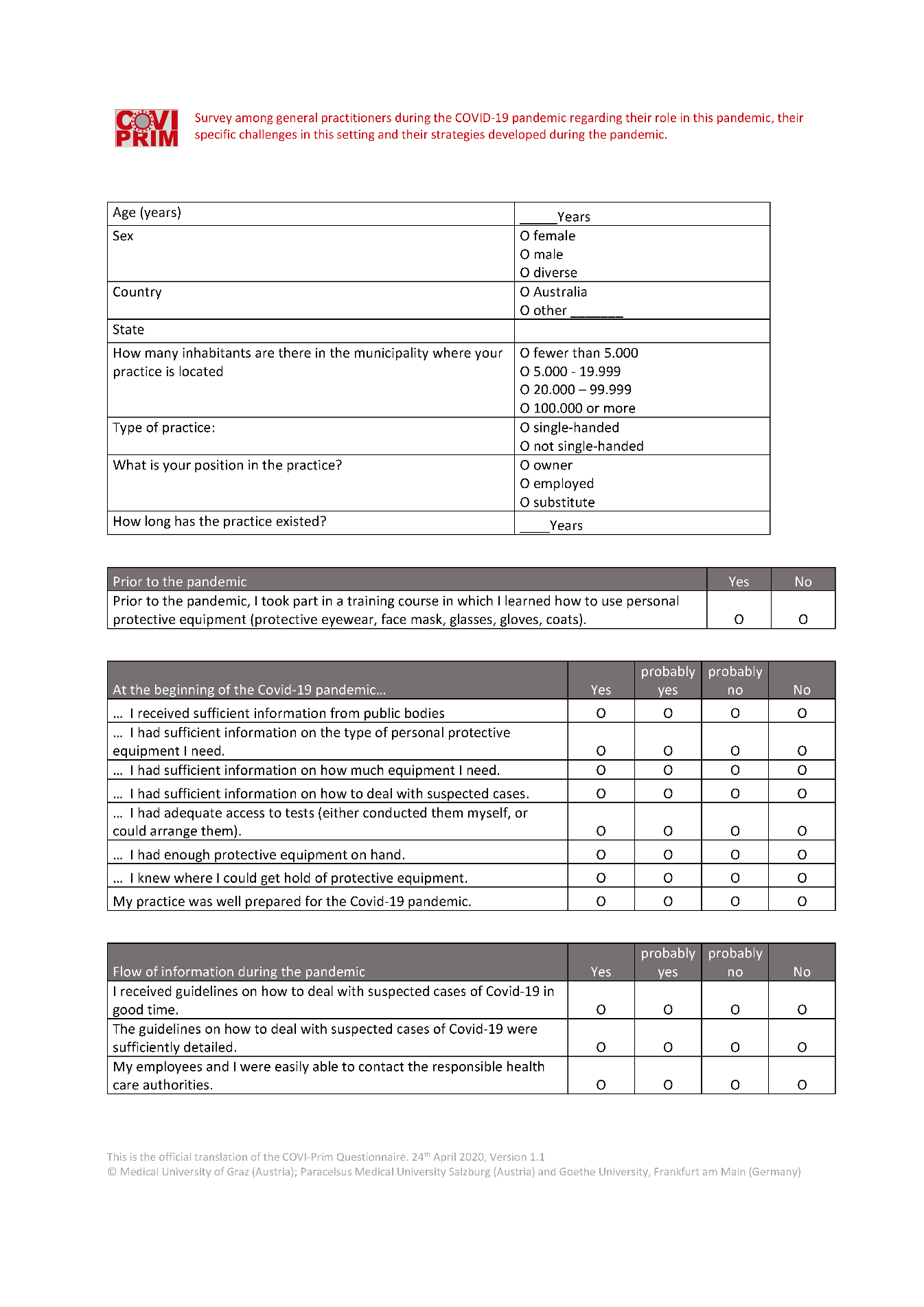

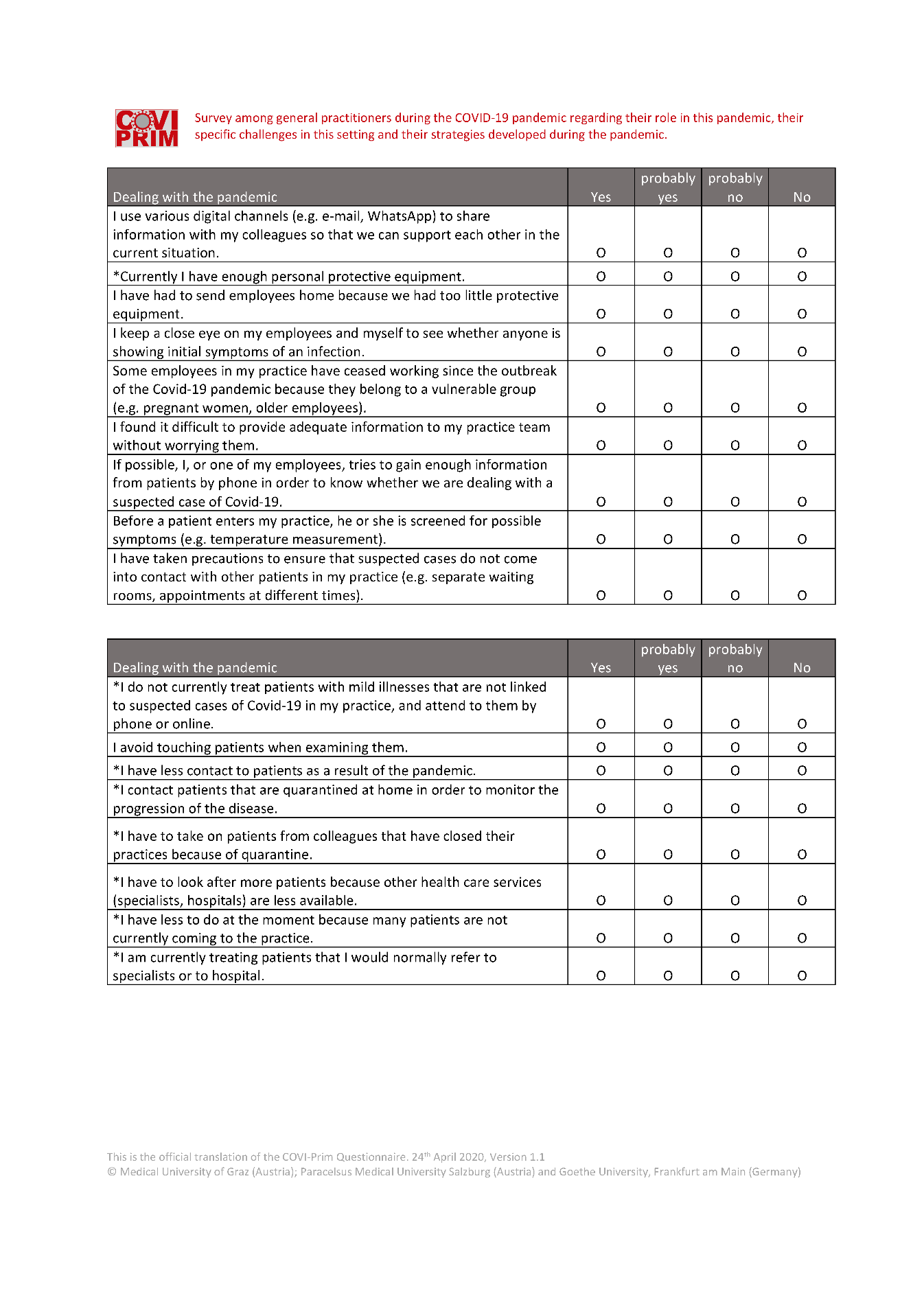

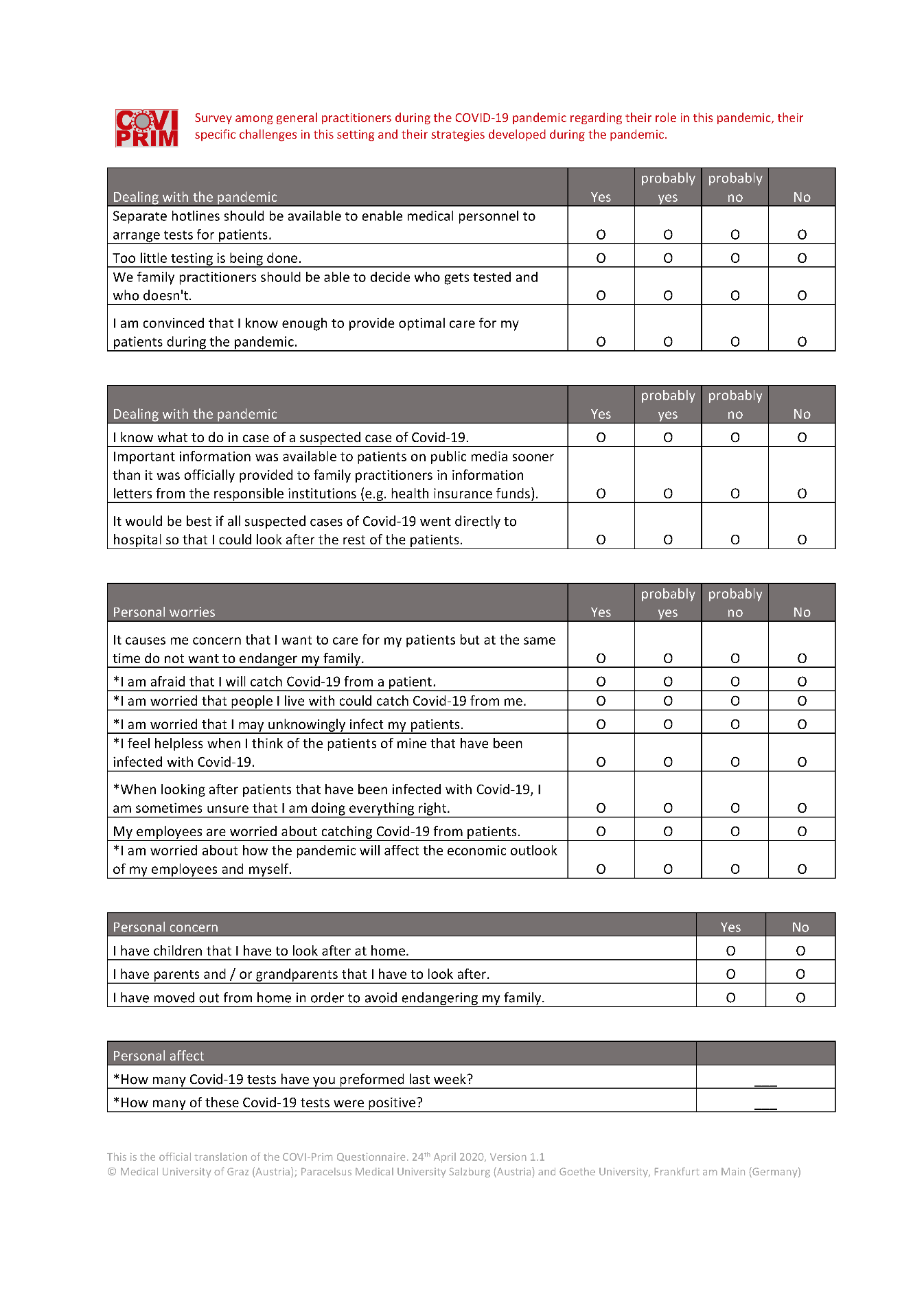

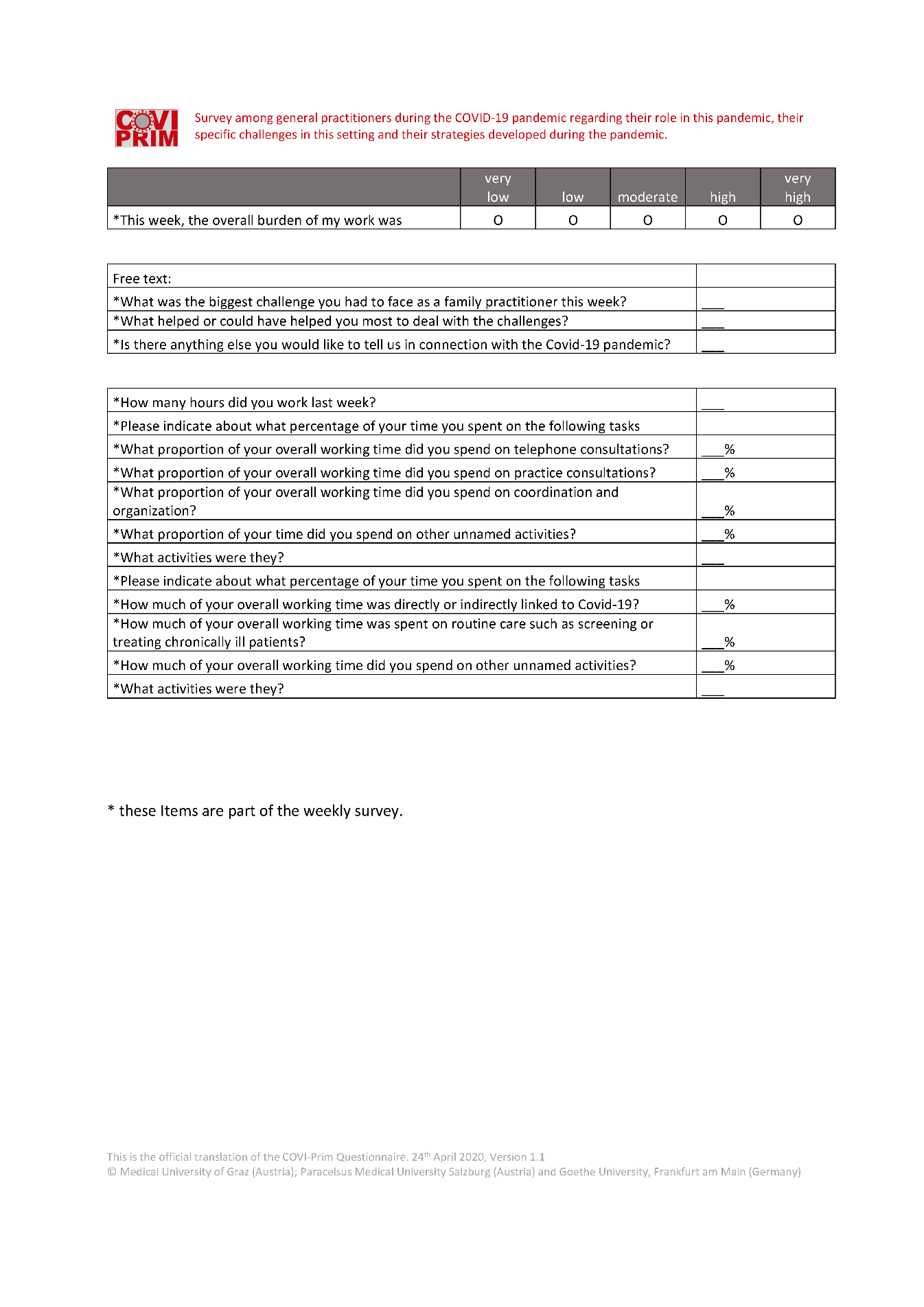

Supplement: S3 File — (DOCX) [file pone.0251736.s007.docx]

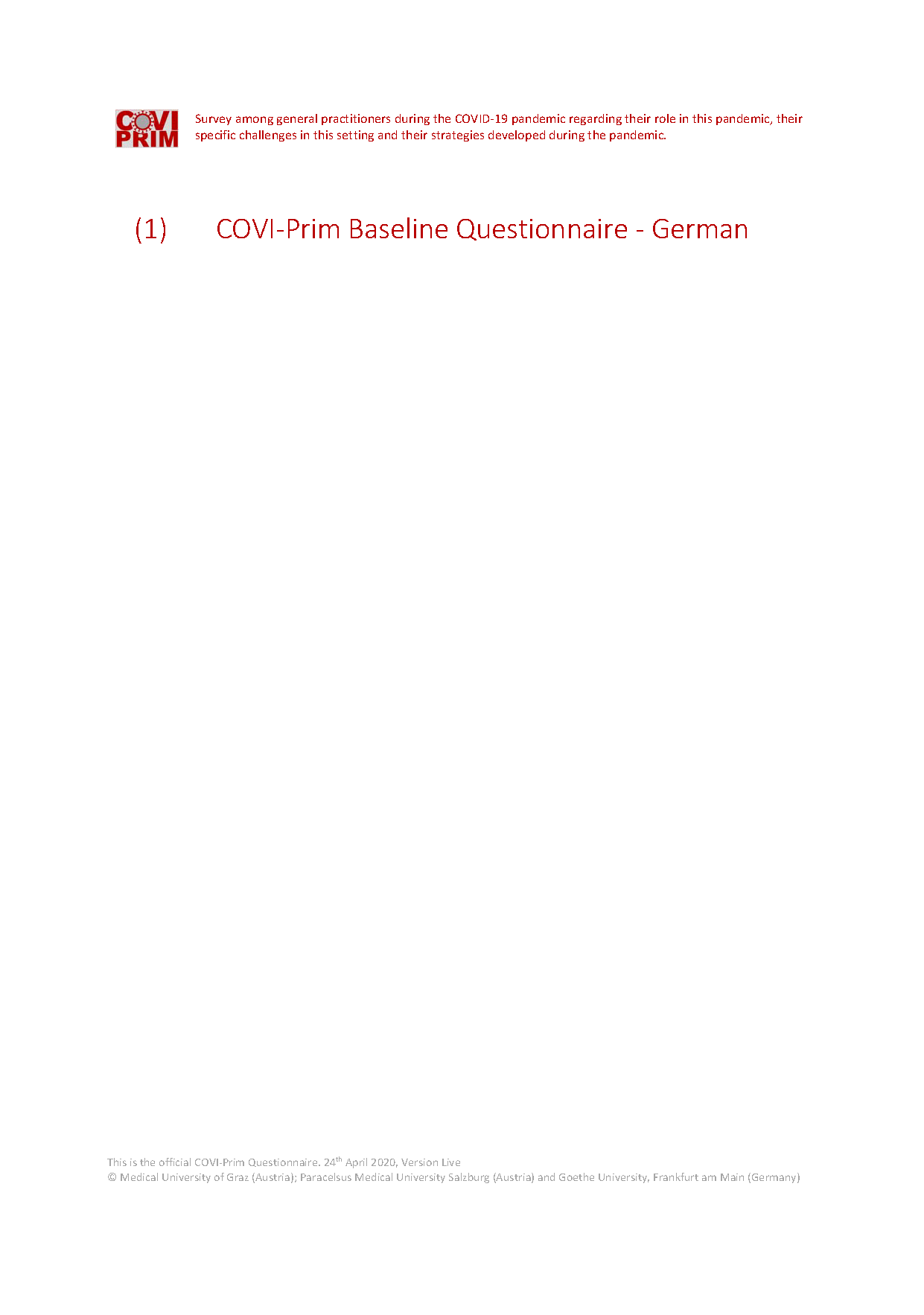


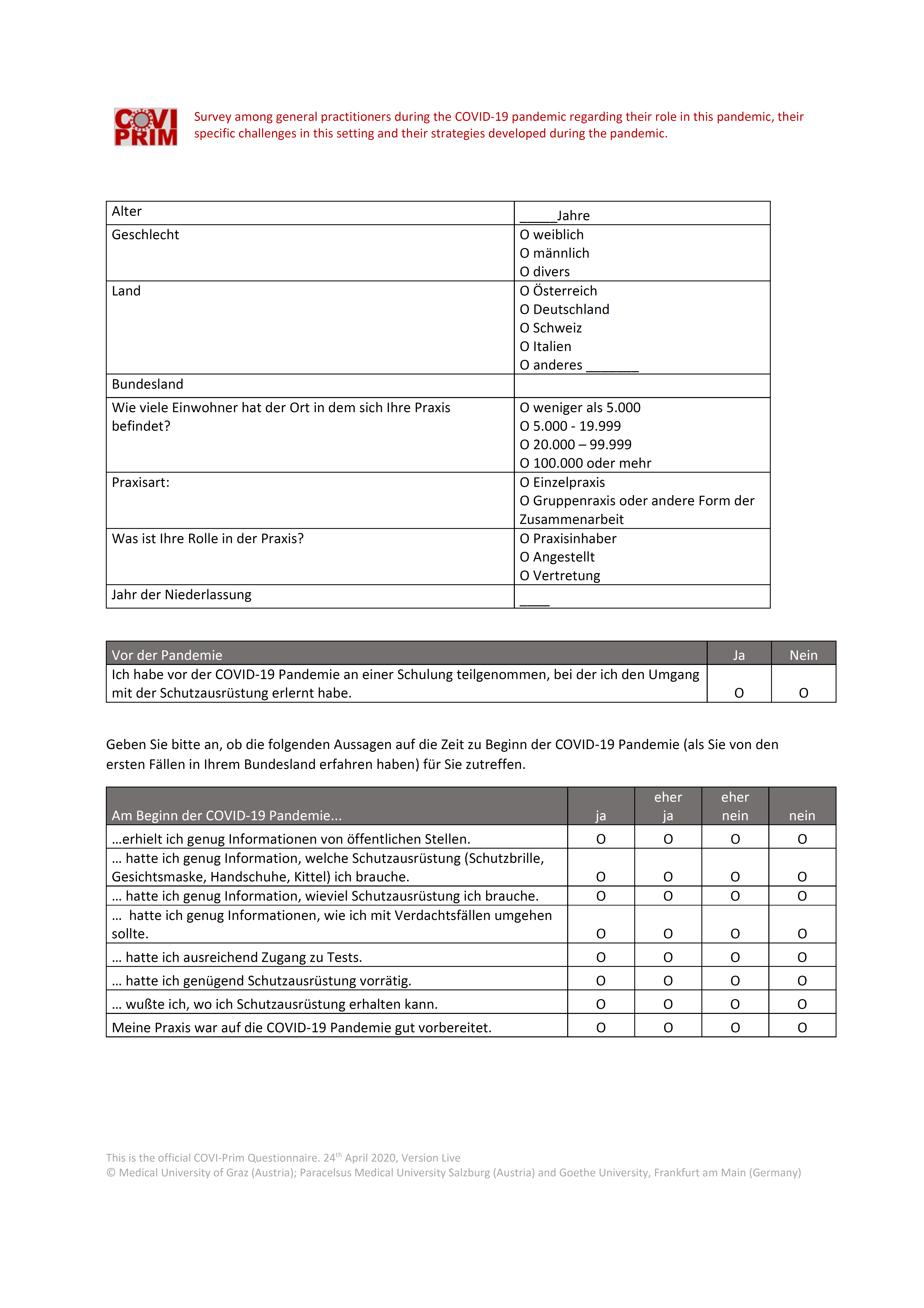

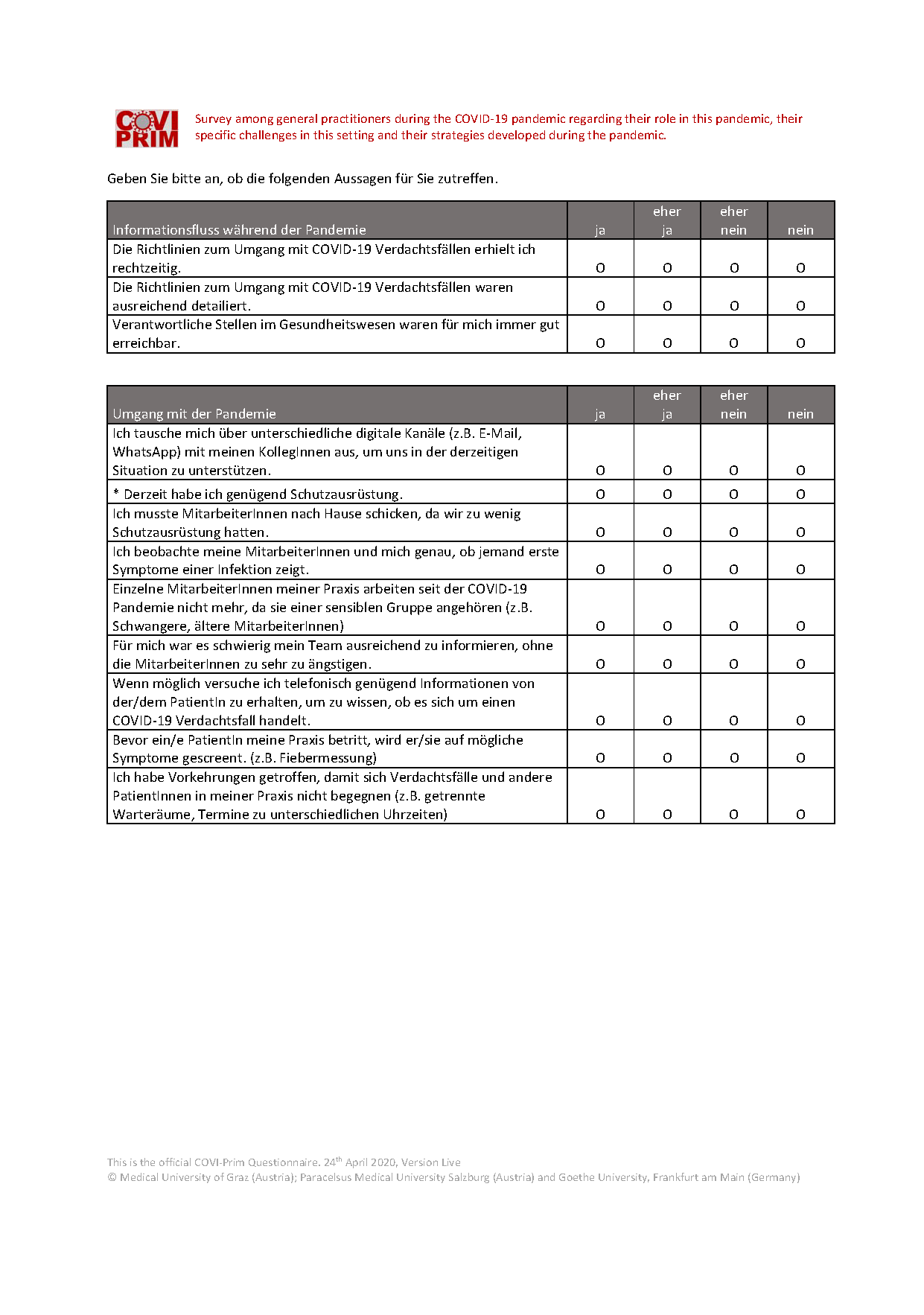

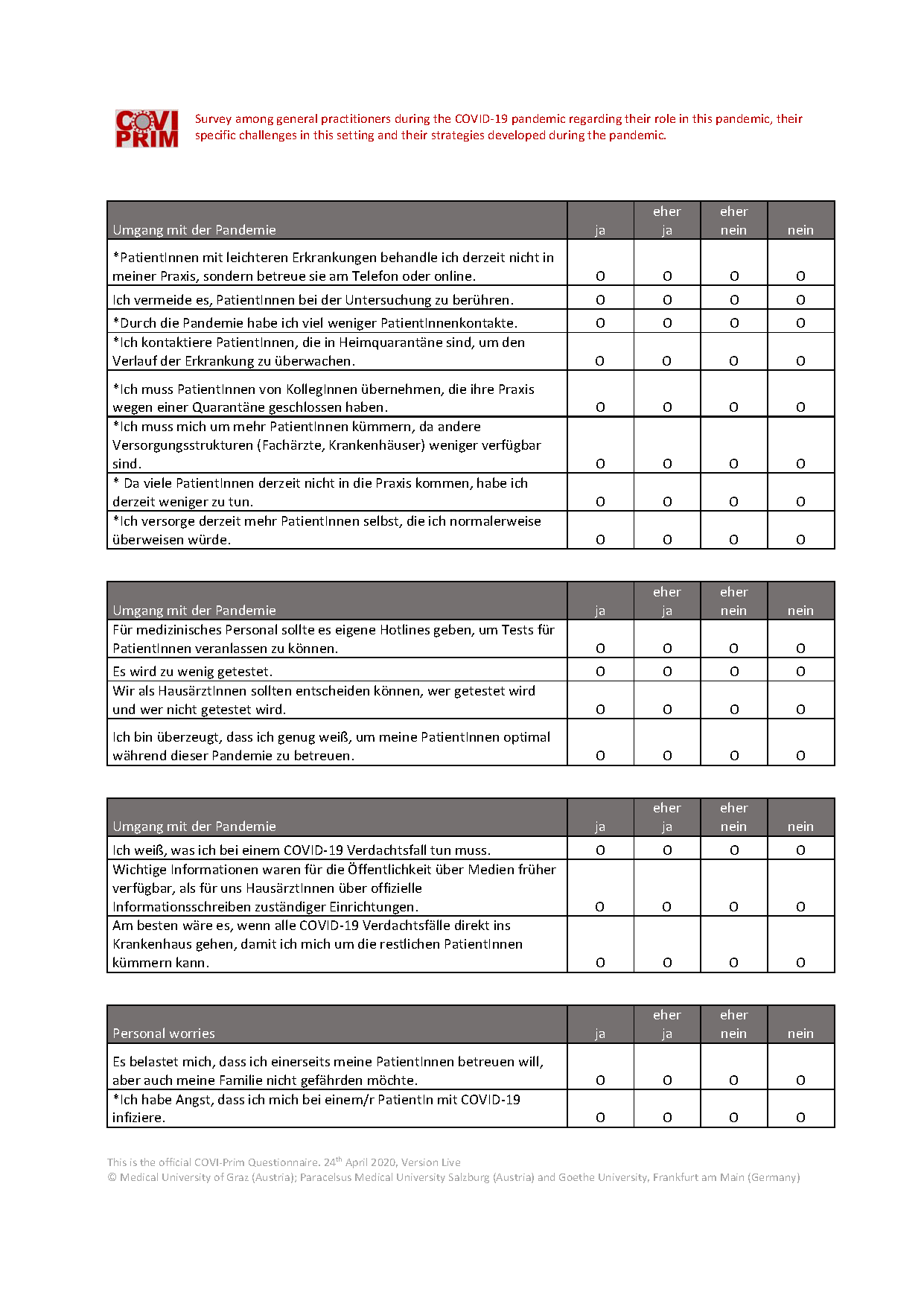

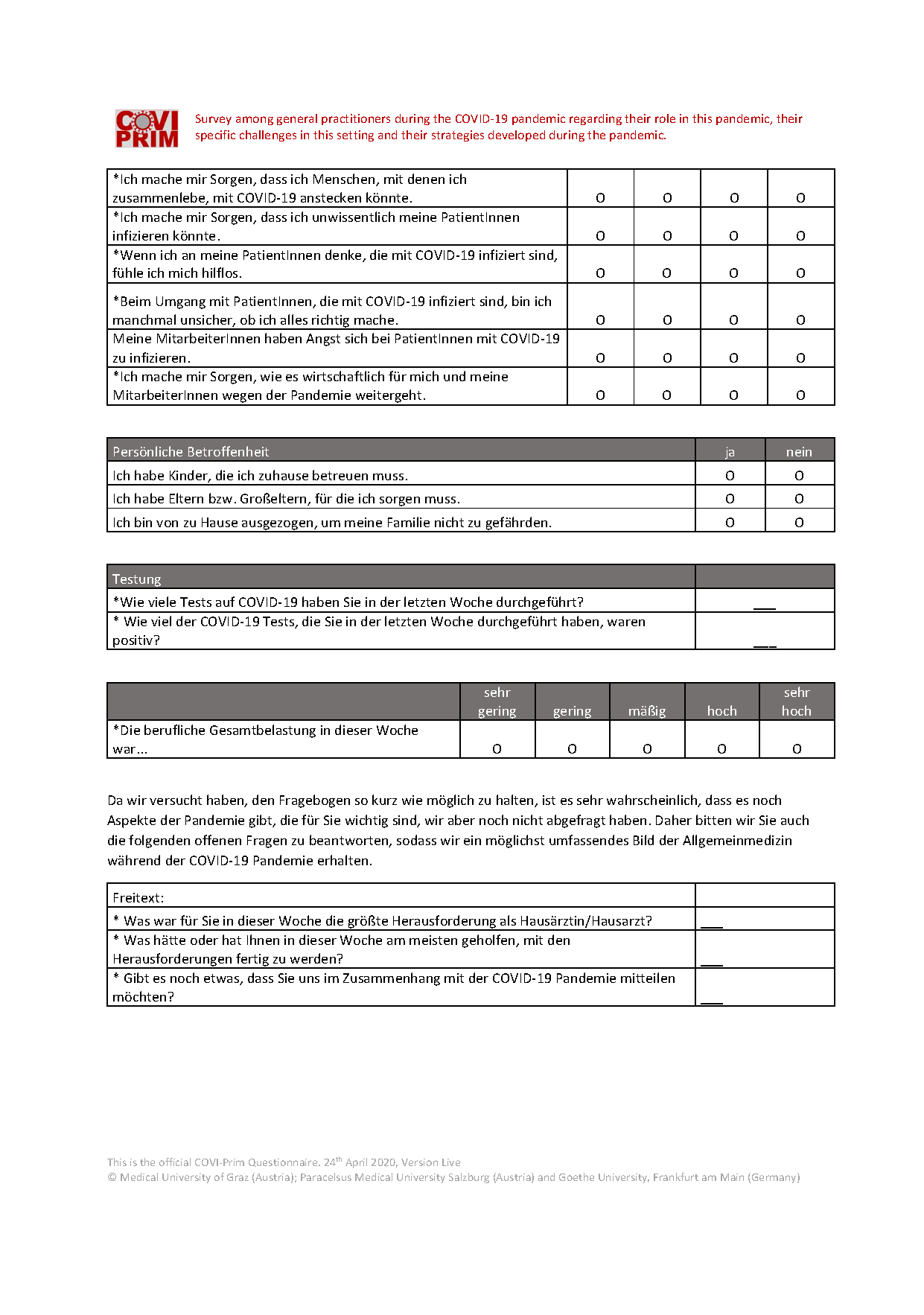

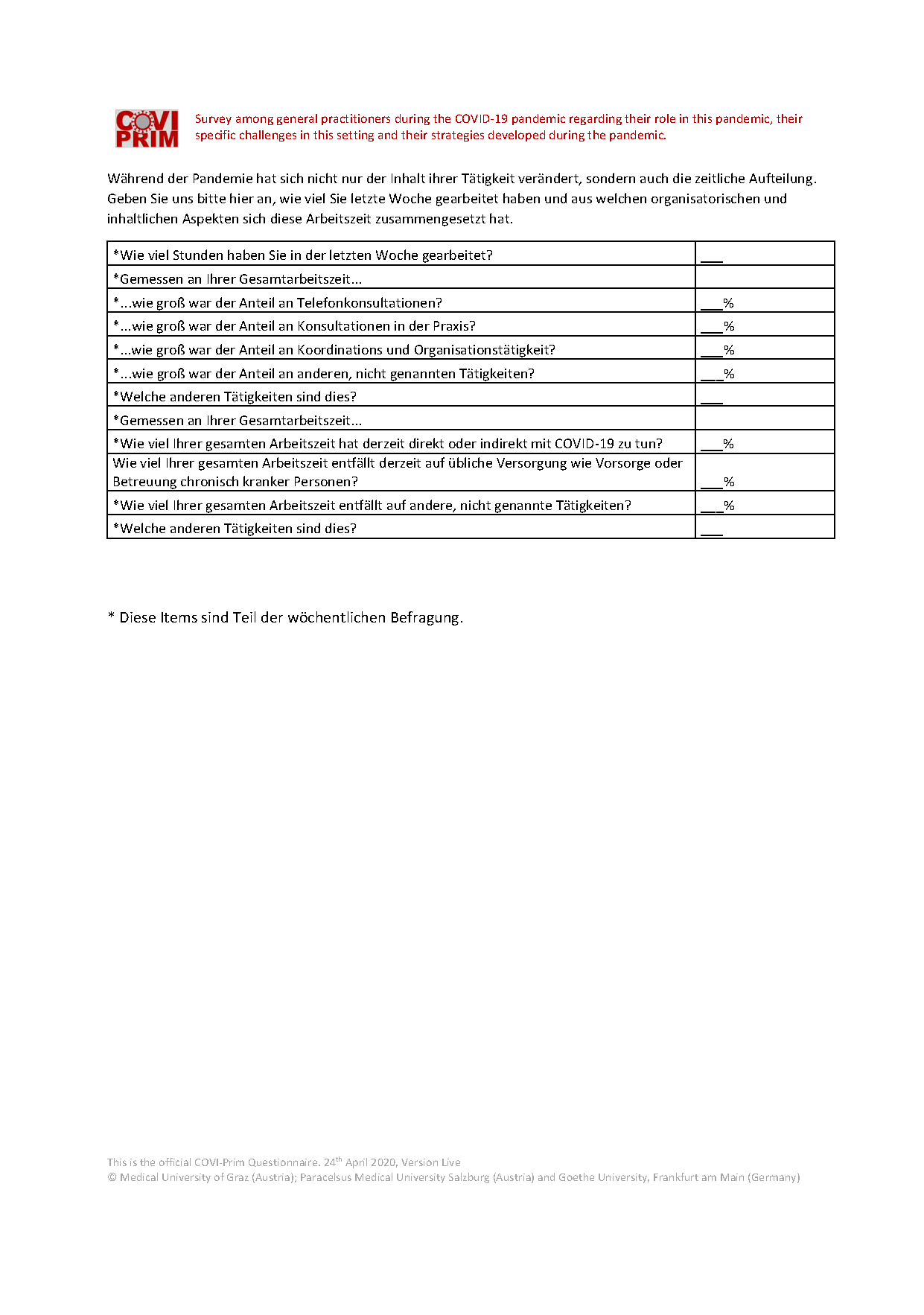

Supplement: S4 File — (DOCX) [file pone.0251736.s008.docx]
