# Supplementary material for: COVI-Prim survey: Challenges for Austrian and German general practitioners during initial phase of COVID-19
Source: PLoS One. 2021 Jun 10;16(6):e0251736. doi: 10.1371/journal.pone.0251736 (PMC8191874; doi:10.1371/journal.pone.0251736)
Supplement: Attachment — Submitted filename: Revision_PLOS one_ask_aa.docx [file pone.0251736.s009.docx]

Dear Mister Gholipour,

Thank you for your response to the submission of our manuscript. We are very grateful for the opportunity to re-submit it to PLOS ONE.

• You must provide a rational for absence of Ethics approval for Austria as mentioned in appendix table 1 considered as Not Applicable.

Response: When we were asking the head of the local ethic committee at the Medical University Graz for an ethical approval, we were told that according to the Austrian law, this study does not require an ethical approval. This information will be added in the method section in the paragraph Ethics at page 6 and the approval from Germany will be uploaded as a supplement file.

• The result of open-end question (free text in questionnaire) was not presented in manuscript. It appears the most related part of questionnaire to your manuscript title was this section, “biggest challenge, supporting factor”.

Response: Prior to the writing of our manuscript we were discussing this aspect in detail and decided that we do not include the comments. This decision was based on the high number (more than 3.500 comments) and the heterogeneity of the comments. We observed that comments differed over time and were strongly dependent of the region of the responding doctors. In our opinion, it is not possible to report these results in one paragraph. We are currently preparing a second manuscript reporting on the interesting results to present the comments in detail.  This information can be added in our actual manuscript for PLOS ONE.

• The results of last table in questionnaire were not presented at all. And were not analyzed in relation to other factors.

Response: We have not analyzed this items in the first version of the manuscript, because, these items do not belong to one of the dimensions of the questionnaire. However, based on your suggestion, we now added the most important results in the manuscript and the other results in the appendix (table S5)

• Considering the unity of covi-prim project please clarify your rational to publish the result of this project separately.

Response: Considering the number of different aspects of the COVI-Prim project, we think that it is not possible to present all results within one manuscript. There are so many aspects (changes over time, differences between countries, differences in baseline between GPs answering the first time at the beginning of the pandemic and GPs answering at the beginning of the summer, a.s.o.) that each of these aspect could be described in several further manuscripts. Therefore, we decided to write one “starter” manuscript including all protocol relevant information in the main text and the supplement that we would be able to reference it within every subsequent publication.

Regarding the funding statement within in the manuscript: We have deleted this sentence. Since we received no funding, there is no need to change the funding statement.

Regarding data availability: We have to apologize, that we missed to provide the DOI. Please insert the following DOI to the data availability statement: <https://doi.org/10.17026/dans-z8u-unbv>

We hope the revised version of our paper is now suitable for publication in your esteemed Journal and look forward to hearing from you soon.

Yours sincerely,

Andrea Siebenhofer
